# Les manifestations cutanées chez les patients hémodialysés chroniques dans un pays en voie de développement

**DOI:** 10.11604/pamj.2016.24.110.8639

**Published:** 2016-05-31

**Authors:** Gérard Coulibaly, Nina Korsaga-Somé, Dorisse Fernade Yongué Fomena, Yacouba Nagalo, Adama Roger Karambiri, Alban Bassolet, Hyacinthe Kafando, Adama Traoré, Adama Lengani

**Affiliations:** 1 Service de Néphrologie et Hémodialyse, Ouagadougou, Burkina Faso; 2 Université de Ouagadougou, Burkina Faso; 3 Service de Dermatologie-Vénérologie, Centre Hospitalier Universitaire Yalgado Ouédraogo, Ouagadougou, Burkina Faso

**Keywords:** Manifestations cutanées, hémodialysés chroniques, Burkina Faso, Skin manifestations, chronic hemodialysis, Burkina Faso

## Abstract

Le but de cette étude était deconnaître les affections cutanées les plus fréquentes chez les patients hémodialysés chroniques du Centre Hospitalier Universitaire Yalgado Ouédraogo (CHU-YO) de Ouagadougou. L’étude, de type transversal descriptif, menée du 15 Septembre au 31 Décembre 2014, s'estdéroulée au CHU-YO. Elle concernait les patients qui avaient au moins trois mois d'ancienneté en hémodialyse chronique. La fréquence des séances d'hémodialyse était d'une tous les cinq jours. Le seuil de signification des tests statistiques était défini pour une probabilité p ≤ 0,05. Quatre-vingt-quinze patients (61,1% d'hommes et 38,9% de femmes), de moyenne d’âge 42,1 ans participaient à l’étude. La durée moyenne en hémodialyse était de 31,9 mois. Le taux de réalisation des analyses biologiques variait de 7,4 à 85,3%. Quatre-vingt patients (85,3%) avaient au moins une manifestation cutanée. La xérose cutanée (67,4%), le prurit (45,3%), et l'hyperpigmentation (23,2%) étaient les plus fréquentes des manifestations cutanées pouvant être spécifiques de l'hémodialyse. L'hypomélanose en gouttes (11,6%), le prurigo (11,6%) et la folliculite (8,4%) étaient les principales manifestations cutanées non spécifiques. L'atteinte cutanée était fréquente mais ne semblait pas liée à l'ancienneté en hémodialyse. Les mauvaises conditions d'hémodialyse et l'environnement sec et chaud à Ouagadougou, ont pu favoriser ces atteintes, en particulier la xérose et le prurit. Une meilleure subvention des soins de santé contribuerait à réduire la prévalence des maladies cutanées et à améliorer la qualité de vie de nos patients hémodialysés chroniques.

## Abstract (en)

Our study aims to highlight the most common skin disorders in patients on chronic hemodialysis at the University Hospital Yalgado Ouédraogo (CHU-YO) in Ouagadougou. The study, of transverse type descriptive, carried out of September 15 to December 31, 2014, is unrolled with the CHU-YO. This descriptive transversal study was conducted at the CHU-YO from September 15 to December 31, 2014. It involved patients who had been on chronic dialysis for at least 3 months. The frequency of hemodialysis sessions was one every five days. The significance level of statistical tests was defined as the probability p ≤ 0.05. Eighty-five patients (61.1% men and 38.9% women) with an average age of 42.1 years were included in the study. The mean duration of hemodialysis was 31.9 months. The success rate of biological examinations varied from 7,4 to 85,3%. Eighty patients (85,3%) had at least one cutaneous manifestation. Cutaneous xerosis (67.4%), pruritus (45.3%), and hyperpigmentation (23.2%) were the most frequent skin manifestations that may be specific of hemodialysis. Guttate hypomelanosis (11.6%), prurigo (11.6%) and folliculitis (8.4%) were the main non-specific skin manifestations. Skin involvement was frequent but did not seem related to seniority in hemodialysis. In Ouagadougou, bad hemodialysis conditions and a hot, dry environment promote such conditions, especially xerosis and pruritus. A better subvention of health care could help to reduce the prevalence of skin diseases and to improve the quality of life of our patients on chronic hemodialysis.

## Introduction

Le patient hémodialysé est sujet à une morbidité et une mortalité élevées. Cela peut être le fait de l'insuffisance rénale chronique (IRC), de l'hémodialyse elle-même, et/ou des comorbidités. Les affections cutanées sont parmi les plus fréquentes et affectent négativement la qualité de vie du patient hémodialysé [1, 2]. Elles sont connues dépressiogènes [3, 4]. La prévalence des manifestations cutanées au cours de l'IRC et chez le sujet hémodialysé est supérieure à 50% [1, 5]. Le prurit est de loin le plus fréquent (50 à 90% des hémodialysés) [6, 7]. Sa physiopathologie est mal élucidée dans la plupart des cas et son traitement difficile [6, 7]. Les études sur les manifestations cutanées chez l'hémodialysé chronique sont rares dans les pays d'Afrique subsaharienne. Au Mali, une étude sur le prurit montrait qu'en milieu dermatologique, les causes systémiques de prurit étaient moins fréquentes et que 8,3% de ces patients avaient une insuffisance rénale [8]. Au Burkina Faso, aucun travail de recherche sur ce sujet n'a encore été mené. Pourtant, nous constatons dans l'unique unité d'hémodialyse du pays, une demande élevée de prise en charge dermatologique. Par la présente étude, nous voulons connaître les affections dermatologiques les plus fréquentes chez les patients hémodialysés chroniques du Centre Hospitalier Universitaire Yalgado Ouédraogo (CHU-YO) de Ouagadougou, afin de contribuer à définir des axes pour leur prévention.

## Méthodes

Notre étude, de type transversal descriptif, s'est déroulée dans l'unité d'hémodialyse et dans le service de Dermatologie-Vénéréologie du CHU-YO de Ouagadougou au Burkina Faso. Elle concernait les patients en hémodialyse chronique de l'unité d'hémodialyse du service de Néphrologie et Hémodialyse du CHUYO. La collecte des données s'est déroulée du 15 Septembre au 31 Décembre 2014, soit sur quatre mois. Nous avons inclus tous les patients hémodialysés qui acceptaient de participer à l’étude et qui avaient une ancienneté en hémodialyse supérieure ou égale à trois mois. Ils réalisaient dans la mesure du possible un bilan biologique sanguin (urée, créatinine, ionogramme, numération formule sanguine) sur un prélèvement avant la dialyse. Ce bilan entrait dans le cadre du suivi de routine du patient hémodialysé. Les définitions usuelles étaient utilisées pour les signes ou affections dermatologiques trouvés. Pendant la période de l’étude, la durée de la séance d'hémodialyse était de cinq heures. La fréquence d'une séance était de tous les cinq jours, soit sept heures d'hémodialyse par semaine, par patient. La teneur en calcium du bain de dialyse était de 1,75 mmol/L. Le circuit utilisé ainsi que les lignes étaient stérilisés à l'oxyde d’éthylène. La membrane d'hémodialyse était le polysulfone. Quatre patients sur 237 étaient traités avec de l’érythropoïétine recombinante. Les patients étaient examinés entre deux séances d'hémodialyse par un dermatologue à l'unité d'hémodialyse ou dans le service de dermatologie. Il n'existait pas de système d'assurance-santé pour les patients hémodialysés de l'unité. Les frais de santé étaient donc entièrement à leur charge. Nous avons recueilli les données sociodémographiques, les antécédents pathologiques personnels, les données cliniques et biologiques. Les données étaient enregistrées puis analysées à l'aide du logiciel Epi-info dans sa version 3.5.3. Pour la comparaison des variables qualitatives, le test de Khi-carré et le test exact de Fischer étaient utilisés. Le test t de Student servait à la comparaison des variables quantitatives. Le seuil de signification statistique était défini pour une probabilité p = 0,05.

## Résultats

### Les caractéristiques générales des patients

Au moment de l’étude, 237 patients étaient en hémodialyse chronique. Parmi eux, 95 (40,1%) participaient à l’étude. **L’âge** moyen des patients était de 42,1 ± 12,4 ans (17-71). Il était de 40,3 ±12,5 ans pour les hommes et de 45 ± 11,8 ans pour les femmes. La différence d’âge entre les hommes et les femmes était à la limite de la significativité (p = 0,05). La répartition selon **le sexe** était de 58 hommes et 37 femmes. **Sur le plan des antécédents**, 25 patients avaient eu une dermatose avant la mise en hémodialyse. La plus fréquente était le pityriasis versicolor (7 cas). Dix patients avaient un antécédent d'allergie médicamenteuse et deux un antécédent d'allergie alimentaire. Sept patients étaient connus diabétiques et 93 hypertendus. Aucun patient n'avait un antécédent de maladie auto-immune. **La durée moyenne en hémodialyse** était de 31,9 ± 30 mois (5-151). **La moyenne de la pression artérielle** était de 162,7 ± 18,5 (111-210) pour la systolique et 91,6 ± 15,4 mm Hg (55-127) pour la diastolique. **Le taux de réalisation** des analyses biologiques variait de 7,4 à 85,3%. Leurs résultats figurent dans le Tableau 1. La calcémie était disponible chez 63 patients avec un taux normal dans 55,6% des cas, une hypocalcémie dans 34,9% des cas et une hypercalcémie dans 9,5% des cas. Quant à la phosphatémie, elle était disponible chez 62 patients avec un taux normal dans 48,4% des cas, une hypophosphatémie dans 17,7% des cas et une hyperphosphatémie dans 33,9% des cas.

**Tableau 1:** Répartition des examens biologiques prescrits aux patients, selon le taux de réalisation, la moyenne et les extrêmes des valeurs obtenues

	n (%)	Moyenne ± DS	Extrêmes
**Créatininémie** (µmol/L)	66 (69,5)	1214,2 ± 386,5	312 – 2110,9
**Uréeplasmatique**(mmol/L)	65 (68,4)	22,2 ± 8,4	4,9 – 53,3
**Calcémie** (mmol/L)	63 (66,3)	2,2 ± 0,3	1,2 – 3,3
**Phosphorémie**(mmol/L)	62 (65,3)	1,4 ± 0,6	0,5 – 3,4
**Kaliémie** (mmol/L)	67 (70,5)	4,9 ± 1,2	2,1 – 8,5
**Bicarbonates** (mmol/L)	55 (57,9)	19,4 ± 3,5	13 – 29
**Protidémie**(g/L)	65 (68,5)	74,9 ± 8,6	49 – 92
PTHi*(pg/mL)	09 (09,4)	551,9 ± 285,1	182,9 – 964,2
ASAT**(UI/L)	07 (07,4)	16,8 ± 4,1	10 – 21,8
ALAT***(UI/lL)	07 (07,4)	12,5 ± 2,6	9 – 15
**Hémoglobinémie** (g/dL)	81 (85,3)	7,4 ± 1,6	3,7 – 12,4
**Leucocytes** (/mm^3^)	81 (85,3)	5510,4 ± 2011,1	1600 – 10800
**Plaquettes** (/mm^3^)	81 (85,3)	219728,4 ± 160838,6	51.10^3^ – 1406.10^3^

* Parathormoneintacte

** Alanine aminotransférase

*** Aspartate Aminotransférase

### Les manifestations cutanées

**Figure 1:**
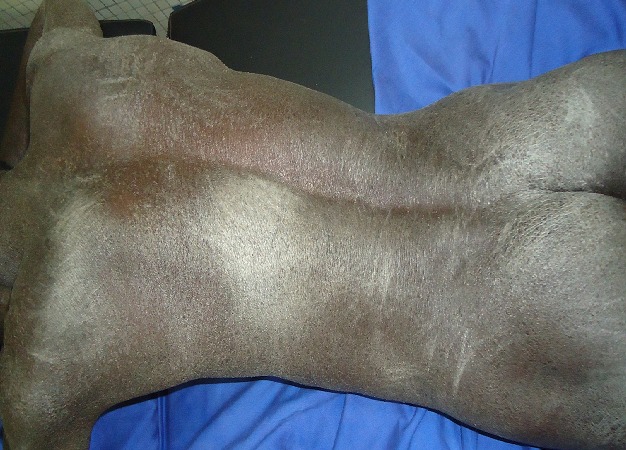
Xérose cutanée diffuse. (Source: collection service de Dermatologie-Vénéréologie du CHU Yalgado Ouédraogo de Ouagadougou)

**Figure 2:**
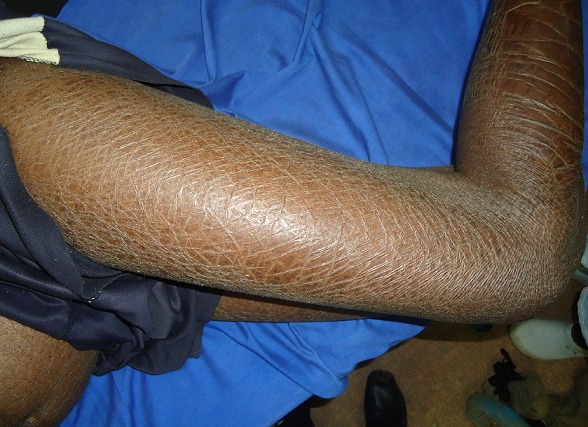
Erose cutanée avec squames ichtyiosiforme. (Source: collection service de Dermatologie-Vénéréologie du CHU Yalgado Ouédraogo de Ouagadougou)

Au moins une manifestation cutanée était présente chez 85,3% des patients. Nous avons distingué les signes cutanés pouvant être spécifiques de l'IRC et/ou de la dialyse elle-même (106) de ceux qui ne l’étaient pas (72). Parmi les manifestations cutanées pouvant être spécifiques de l'IRC et/ou de la dialyse elle-même, la xérose cutanée, le prurit et l'hyperpigmentation étaient les plus fréquents. Nous avons noté 64 cas de **xérose cutanée**. Elle était diffuse (Figure 1) chez 5 patients et localisée chez 59 patients. Elle se présentait sous la forme d'un aspect rugueux de la peau au toucher chez 47 patients, de squames pityriasiformes chez 10 patients et de squames ichtyosiformes (Figure 2) chez 2 patients. Cette xérose cutanée était prurigineuse dans 50% des cas. La deuxième manifestation cutanée qui était **le prurit** était présent chez 45,3% (43) des patients. Ce prurit était diffus chez 35 patients et localisé au dos et/ou aux membres chez 8 patients. Il survenait pendant la séance de dialyse chez 29 patients, était permanent chez 11 patients, et apparaissait entre deux séances de dialyse chez 3 patients. Son intensité était variable, et il entrainait des stries de grattage et de la lichénification dans les formes sévères. La troisième manifestation cutanée en terme de fréquence était **l'hyperpigmentation** qui était retrouvée chez 22 patients (23,2%). Elle prédominait sur les zones photo-exposées. Les autres signes cutanés pouvant être spécifiques de l'IRC et/ou de la dialyse elle-même sont représentés dans le Tableau 2. Le Tableau 3 présente les manifestations cutanées non spécifiques de l'IRC et/ou de la dialyse elle-même. Nous n'avons pas trouvé de lésions d'amylose cutanée, de calcification cutanée, de dermatose perforante acquise, de calciphylaxis ni de fibrose néphrogénique systémique.

**Tableau 2:** Répartition des manifestations cutanées pouvant être spécifiques de l'insuffisance rénale chronique et/ou de l'hémodialyse selon la fréquence

	Effectif	Pourcentage
Xérose cutanée	64	67,4
Prurit	43	45,3
Hyperpigmentation	22	23,2
Chute de cheveux	11	11,6
Xanthonychie	5	5,3
Mélanonychie	2	2,1
Ongles équisegmentés	1	1,1
Ecchymose	1	1,1

**Tableau 3:** Répartition des manifestations cutanées non spécifiques de l'insuffisance rénale chronique et/ou de l'hémodialyse selon la fréquence

	Effectif	Pourcentage
Hypomélanose en gouttes	11	11,6
Prurigo	11	11,6
Folliculite	8	8,4
Acné	7	7,4
Intertrigo des espaces inter orteils	6	6,3
Chéloïdes sur la FAV*	5	5,3
Kératose folliculaire	4	4,2
Lymphœdème de membre	3	3,2
Pityriasis versicolor	3	3,2
Ulcère du dos du pied	3	3,2
Dermatophytie	3	3,2
Erysipèle de jambe	2	2,1
Hyperkératose plantaire	2	2,1
Dermite séborrhéique	1	1,1
Dermite d'irritation**	1	1,1
Eczéma	1	1,1
Vitiligo	1	1,1

^+^Fistuleartérioveineused'hémodialyse. ^++^Lié à l'usage de sparadrapperforéutilisé pour le pansement du cathéterd'hémodialyse

## Discussion

Les patients qui avaient des antécédents d'allergie ou de prurit n’étaient pas évalués sur le plan allergologique du fait du coût élevé des tests allergologiques. Le bilan biologique était assez restreint compte tenu des moyens financiers limités de nos patients. Le taux de participation était également lié au manque de ressources financières chez la plupart des patients. Malgré tout, nous croyons avoir atteint notre objectif en obtenant des données initiales fiables sur la pathologie dermatologique du patient hémodialysé chronique à Ouagadougou. La fréquence des manifestations cutanées au cours de l'IRC était élevée dans notre série. Dans la littérature, elle varie de 50 à 100% [8–10]. Il n'y avait pas de lien apparent entre la survenue de manifestations cutanées chez nos patients et leur ancienneté en hémodialyse. Mourad et coll en Égypte [11] et Udayakumar et coll en Inde [12] faisaient le même constat. Une fréquence élevée de xérose cutanée était aussi rapportée par d'autres auteurs avec des taux pouvant atteindre 96% [5, 12–15]. Par contre Hajheydari et Makhlough [16] trouvaient un taux curieusement plus faible (23%), mais leur étude était réalisée dans une région particulièrement humide. La pathogénie de la xérose est encore inconnue. Les facteurs habituellement mis en cause sont le traitement diurétique à des doses élevées, l'hypervitaminose A, l’élévation du taux de retinolbindingprotein, l'alcalinité de la peau, la malnutrition protéino-énergétique secondaire à un régime diététique sévère, la carence en glycérol avec déshydratation de la peau, le dysfonctionnement de la barrière, l'irritation induite chimiquement et les anomalies fonctionnelles des glandes sudoripares eccrines [17–20]. L'environnement sec et souvent chaud pendant les trois quarts de temps de l'année dans la ville de Ouagadougou pourrait être un facteur supplémentaire favorisant la xérose.

**Le prurit** est un signe cutané fréquent chez le sujet hémodialysé chronique. La prévalence que nous avons trouvé était comparable à celle de Sayed et coll (43%) [21]. Masmoudi [22] notait un prurit amélioré par la dialyse dans 18,9% des cas, et exacerbé dans 35,6% des cas. La différence entre les résultats de Masmoudi [22] et les nôtres concernant la fréquence du prurit pourrait être attribuée aux conditions d'hémodialyse moins bonnes dans notre contexte. En effet au cours de notre étude, la fréquence des séances d'hémodialyse était d'une tous les cinq jours. De plus, le circuit était rincé avec peu de sérum physiologique dans un souci d’économie de ressources. Le risque de réaction allergique aux composantes du circuit pourrait donc être élevé dans notre population. En prenant en compte les résultats de Hiroshige [23], une dialyse adéquate ainsi qu'une alimentation équilibrée contribueraient significativement à la réduction de la prévalence du prurit chez nos patients. Il convient de rappeler que la physiopathologie du prurit chez l'hémodialysé reste encore mal élucidée. Plusieurs facteurs interviendraient, notamment l’élévation des taux sériques de phosphore, de calcium, de magnésium, d′histamine, l'hyposidérémie et la xérose cutanée [24, 25]. Les taux habituellement rapports **d'hyperpigmentation** dans la littérature sont extrêmement variés, allant de 17 à 94% [5, 9, 26]. Notre série était entièrement constituée de patients à peau noire. L'hyperpigmentation a pu alors passée inaperçue dans certains cas. Elle est attribuée à une augmentation de la mélanine dans la couche basale. Cette augmentation serait due à une excrétion insuffisante de ß-MSH par les reins et la dialyse [15]. L′hémodiafiltration permet d′augmenter la clairance de ß-MSH et d'améliorer ainsi l'hyperpigmentation [27].

## Conclusion

Les manifestations cutanées sont fréquentes et variées chez nos patients hémodialysés chroniques dans un contexte d'insuffisance de ressources. Elles sont représentées essentiellement par la xérose cutanée, le prurit et l'hyperpigmentation. L'augmentation des ressources humaines et matérielles, l'amélioration du cadre de vie des patients ainsi qu'une meilleure subvention des soins de santé contribueraient à réduire la fréquence des maladies cutanées et à améliorer la qualité de vie de nos patients hémodialysés chroniques.

### Etat des connaissances actuelles sur le sujet

- Les maladies cutanées peuvent être source de dépression;
- La prévalence des manifestations cutanées est élevée chez le sujet hémodialysé chronique;
- Le prurit et la xérose cutanée sont les manifestations cutanées les plus fréquentes dans cette population.

### Contribution de notre étude à la connaissance

- Les données épidémiologiques apportées contribueront à une meilleure connaissance des manifestations cutanées chez le sujet hémodialysé dans les pays en développement où l'on note encore une insuffisance de données rapportées dans la littérature indexée sur le sujet;
- Nous avons confirmé la grande prévalence des manifestations cutanées chez l'hémodialysé chronique;
- Notre étude permet de souligner l'importance d'allier un traitement de qualité à une bonne nutrition des patients; ce dernier aspect n'est pas assez souvent promu, pour des raisons diverses, dans les unités dhémodialyse de cette partie du monde.
